# Distinct clinical phenotypes for Crohn’s disease derived from patient surveys

**DOI:** 10.1186/s12876-021-01740-6

**Published:** 2021-04-09

**Authors:** Tianyun Liu, Lichy Han, Mera Tilley, Lovisa Afzelius, Mateusz Maciejewski, Scott Jelinsky, Chao Tian, Matthew McIntyre, the 23andMe Research Team, Michelle Agee, Adam Auton, Robert K. Bell, Katarzyna Bryc, Sarah L. Elson, Pierre Fontanillas, Nicholas A. Furlotte, David A. Hinds, Karen E. Huber, Aaron Kleinman, Nadia K. Litterman, Jennifer C. McCreight, Matthew McIntyre, Joanna L. Mountain, Elizabeth S. Noblin, Carrie A. M. Northover, Steven J. Pitts, J. Fah Sathirapongsasuti, Olga V. Sazonova, Janie F. Shelton, Suyash Shringarpure, Chao Tian, Joyce Y. Tung, Vladimir Vacic, Nan Bing, Kenneth Hung, Russ B. Altman

**Affiliations:** 1 Department of Bioengineering, Stanford University, Shriram Room 209, MC: 4245, 443 Via Ortega Drive, Stanford, CA 94305-4145 USA; 2 Biomedical Informatics Training Program, Stanford University, Stanford, CA USA; 3 Inflammation and Immunology, Pfizer Inc., Cambridge, MA USA; 4 23andMe Research Team, 23andMe Inc., Sunnyvale, CA USA

**Keywords:** Crohn’s disease, Patient-reported data, Phenotypes, Subtypes, Classification

## Abstract

**Background:**

Defining clinical phenotypes provides opportunities for new diagnostics and may provide insights into early intervention and disease prevention. There is increasing evidence that patient-derived health data may contain information that complements traditional methods of clinical phenotyping. The utility of these data for defining meaningful phenotypic groups is of great interest because social media and online resources make it possible to query large cohorts of patients with health conditions.

**Methods:**

We evaluated the degree to which patient-reported categorical data is useful for discovering subclinical phenotypes and evaluated its utility for discovering new measures of disease severity, treatment response and genetic architecture. Specifically, we examined the responses of 1961 patients with inflammatory bowel disease to questionnaires in search of sub-phenotypes. We applied machine learning methods to identify novel subtypes of Crohn’s disease and studied their associations with drug responses.

**Results:**

Using the patients’ self-reported information, we identified two subpopulations of Crohn’s disease; these subpopulations differ in disease severity, associations with smoking, and genetic transmission patterns. We also identified distinct features of drug response for the two Crohn’s disease subtypes. These subtypes show a trend towards differential genotype signatures.

**Conclusion:**

Our findings suggest that patient-defined data can have unplanned utility for defining disease subtypes and may be useful for guiding treatment approaches.

**Supplementary Information:**

The online version contains supplementary material available at 10.1186/s12876-021-01740-6.

## Background

Understanding how clinical phenotypes emerge from the combination of genotype and environmental exposures is a major challenge. Defining the molecular basis for clinical phenotypes provides opportunities for new diagnostics and new therapies, and may provide opportunities for early intervention and disease prevention. Patient-reported health and medical data are increasingly available and provide potentially useful data for defining disease subtypes. However, the methods for analysis of patient-reported descriptions are still nascent. Patient-reported data can be obtained in many ways ranging from direct patient surveys to mining of social media. In the last decade, there has been great interest in using data from mobile communications and electronic health technology to improve health [1]. Patient-reported data collected from mobile communications and electronic health technology showed agreement with clinical practice and have been successfully used in telemedicine for management of chronic illness [2–8]. However, it remains unclear at what extent to which patient-reported data provides unplanned insights into unknown dimensions of disease heterogeneity and drug responses.

Phenotypic classification is critical in inflammatory bowel disease (IBD) because it is a heterogeneous disease and sub-classifications [e.g. Crohn’s Disease (CD) versus Ulcerative Colitis (UC)] are associated with different therapeutic strategies [9–11]. The definition of novel IBD subtypes may increase the likelihood of finding new susceptibility genes that are specific to those phenotypes. Currently, there are three classification systems for IBD: (1) anatomically based systems that define areas of the gut affected by disease; (2) severity-based measures that generally use clinical symptoms or simple tests to assess the severity of disease; and (3) quality of life measures that generally use patient questionnaires to assess overall well-being and social functioning [12].

In general, IBD is often sub-classified into UC and CD. However, this classification does not always predict treatment response well and there are many patients who do not neatly fit either prototype [13]. Thus, the definition of disease subtypes that are more homogeneous phenotypes remains a challenge. In 1998, the Vienna classification was introduced, which was the first attempt to classify different clinical phenotypes of CD by providing a structured format for a minimal data set on the most important variables [14]. In 2005, the Montreal classification described the extent and behavior of CD in more detail and also included a classification system for UC [15]. The subtype classification of UC is essentially based on disease location, distinguishing proctitis, left-sided colitis and extensive colitis. For CD, clinical sub-phenotypes are based on age at diagnosis, disease location, and behavior [9, 15, 16]. Reliable IBD subtype classification is key for identifying genetic markers that predict disease course [17–20]. Lee et al. have reported that certain genes determine the disease locations and IBD can be classified into three main disease subtypes based on locations. However, they were unable to define genetic variants associated with the subtypes classified by disease behavior individually, suggesting a more refined and integrated classification is needed [20].

Despite these efforts, standardized methods for describing and assigning clinical phenotypes remains a challenge [21]. Information regarding the patient’s age and sex, disease location, and disease progression is needed for patient care. Information on disease location is crucial when considering topical therapy. Information on disease progression is pertinent when considering immune suppressive therapy. In order to translate the complexity of disease characteristics into useful research models for classification, it is important to develop detailed and unambiguous criteria for defining phenotypes. All these information sources are derived from a physician and healthcare system view of the disease and may not correspond to the patient experience. For this reason, there has been interest in seeking patient-derived phenotype definitions to determine if they provide more clarity than the healthcare system-derived phenotypes. The NIDDK IBD Genetics Consortium (IBDGC) collected DNA and phenotypic data from IBD subjects [22]. They surveyed CD patients and ascertained (1) smoking history prior to diagnosis (2) macroscopic disease location (3) surgery (4) extra-intestinal manifestations (joints, skin, eyes, liver). Steinhart et al. [22] assessed the reliability and validity of IBD phenotyping within IBDGC using a standard protocol for phenotypic assessments. They found that the patient-derived phenotypes were in good agreement with those derived from standard clinical protocols (disease location for CD, disease extent for UC, and disease behavior based on Vienna classification). However, a critical question is not whether the patient-reported data replicates healthcare system-based categories, but whether it may provide a better set of criteria for classifying patients.

In this work, we evaluate the degree to which IBD patient-reported survey health and medical data suggests disease sub-phenotypes that are useful for prognosis and therapy selection. Specifically, we identify novel subtypes of CD that discriminate disease severity and show different drug responses. We also report on the hazards and challenges of questionnaire design to help guide future survey activities.

## Methods

### Questionnaire design

**Fig. 1:**
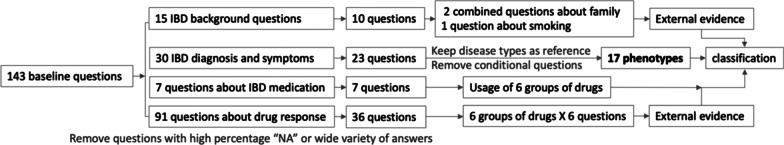
Structure of the questionnaire and the process of question selection. A total of seventeen questions are used for classification. External evidence (or labels) derived from background questions and drug response are used to evaluate the classification. Details of the selection process are in Additional file 1: Table S1 and the “Methods” section

The questionnaire was designed for other research purposes (between Pfizer and 23andMe), including genotyping analysis on IBD patients (unpublished data). The original questionnaire contains 143 baseline questions in four sections: fifteen questions are in the section of IBD Background, 30 questions in the section of IBD Diagnosis and Symptoms, seven general questions about medication in the section of IBD Medications (Fig. 1). In the section of Drug Responses, patients answered seven questions about drug response for each of the thirteen drug groups (salicylates, corticosteroids, antibiotics, two groups of immunosuppressants, nine groups of TNF-inhibitors), resulting in 91 questions. A summary of the questionnaire is available in Additional file 1: Table S1. The complete questionnaire is also available in Additional file 2. The questionnaire collected answers from 5355 patients for the 143 baseline questions. Recruitment and survey responses were collected online and from participants in the United States. The questionnaire has not been validated.

### Select phenotypes and patients from questionnaire

1. Initial selection

From the fifteen questions in the section of IBD Background, we removed five questions because of high percentage of “NA” in their answers. From the 30 questions in IBD Diagnosis and Symptoms, we removed these seven questions: (1) We removed four questions that addresses the year of diagnosis and disease onset because we could not track down the age of diagnosis and the age of disease onset due to the lack of birthdate information. (2) We removed question “In the past year, has your IBD caused any of the following symptoms elsewhere in your body? Please select all that apply”, because patients could select any combinations of the seven symptoms provided, which resulted in wide variety of answers. (3) We removed two questions regarding to fistula because only a very small population has (< 5%) this issue. This led to the 23 questions in IBD Diagnosis and Symptoms. From the original 91 questions (thirteen drug groups) in section of Drug Responses, we removed seven groups, of which only a very low percentage of patients have taken. We analyzed questions for each of the six drug groups to estimate drug efficacy (Fig. 1). Altogether with the seven questions in the section of IBD Medications, a total of 76 questions are renumbered and listed in Additional file 1: Table S1.

2. Phenotypes for classification and external measurements

We combined the ten questions in the section IBD Background into three phenotypes: smoking, maternal inheritance, and fraternal inheritance. These three phenotypes were used as external measurements to evaluate classifications. Questions regarding the six drug groups (salicylates, corticosteroids, immunosuppressants, antibiotics, infliximab and adalimumab) in the sections of IBD Medications and Drug Responses (Additional file 1: Table S1) were used to estimate drug usage and drug efficacy as external measurements to evaluate classifications.

We made use of seventeen questions from the 23 questions in the section of IBD Diagnosis and Symptoms for classification. First, we kept question #11 (What kind of IBD do you have?) as external clinical diagnosis labels, not as phenotype features for classification. Second, when a patient’s answer to question #23 “Have you experienced any skin conditions or mouth sores since being diagnosed with IBD?” is “YES”, the patient is asked to answer a series of questions “Which skin conditions have you experienced?” (#24 to # 28: Erythema nodosum, Pyoderma gangrenosum, Psoriasis, Small ulcers in the mouth, Eczema). Otherwise, patients can skip questions #24 to #28. We removed the five questions (#24-#28) because only a very small number of patients give “YES” to one of these questions, leaving a very large number of “NA” in the data. Therefore, we only collect information of “YES” or “NO” for the phenotype of skin conditions. Last, the question #12 (When was your last IBD flare period?) was used to separate patient groups and was not considered as a phenotype.

3. Patient selection

When answering to the question (#12) “When was your last IBD flare period?”, the answer options are: “Less than 3 months ago”, “3–6 months ago”, “6–12 months ago” and “More than a year ago”. According to answers to question #21, patients were divided into two groups: those who had flares in the last 12 months and those who did not*.* Patients who did not have flares in the last 12 months did not provide answers to the next four questions regarding to the symptoms during a flare, resulting in “NA” as their answers to those questions. In order to effectively generate feature representations for these important phenotypes, we chose patients who had flares in the last 12 months for analysis (“YES” to question #12), resulting in 3576 patients (2140 CD and 1436 UC). We then collected patients who give answers that are not “NA” to all these seventeen questions, resulting in 1961 patients (1118 CD and 843 UC).

### Identify disease subtypes and analyze features

To determine the optimal number of sub-populations within the patients, we used model-based clustering, which uses a penalty function to control the amount of model complexity, thus allowing some flexibility in the shapes of the multivariate normal distribution for values of phenotypic assignments by maximizing a penalized likelihood [23, 24]. Specifically, we used the GMM package in MatLab (Gaussian mixture models using the Expectation–Maximization algorithm) and explored a full range of covariance structures. Patients were clustered into two groups using “full unshared” feature.

We made use of questions in section of IBD Background as external measurements to evaluate classifications based the phenotypes extracted from the section IBD Diagnosis and Symptoms. The significance of feature enrichments within a disease subtype was estimated by calculating the hypergeometric *p* value. We also collected answers to the questions in the section of Drug Responses to evaluate disease subtypes. We analyzed the significance of positive drug response (effective) observed in different CD subtypes by logistic regression and chi square test using R. We fit the drug responses to a simple logistic regression model and carried out a statistical test for associations between CD subtype classification and outcome. We then calculated the estimated odd ratio and confidence intervals.

### Identify genotyping signature

Genotype measurements were conducted by 23andMe Inc (unpublished data). Using PLINK 1.07 [25], we conducted a genome wide association study to assess for significant variants between the CD and UC populations, and subtypes. All variants, measured and imputed, were tested for significance using the Pearson’s chi-square test. Variants were considered significant at the genome wide association significance cutoff of *p* = 1e−8.

## Results

### Phenotypes extracted from patient questionnaires

The original patient survey data provided answers for 143 baseline questions from 5355 patients. In initial analysis, we had to remove 67 questions due to the high percentage of “NA” in the corresponding answers. PCA analysis on the 33 questions in the sections IBD Background and IBD Diagnosis and Symptoms (Fig. 1 and Additional file 1: Table S1) showed feature dependency caused by conditional questions (Additional file 1: Figure S1). Therefore, we compared several strategies for question selection and pruning in order to generate independent phenotypes. We decided on seventeen phenotypes out of the thirty questions (57%) in IBD Diagnosis and Symptoms for classification, three phenotypes (defined by ten questions) out of the fifteen questions (67%) in IBD Background, and questions for six out of the thirteen drug groups (46%) in the sections of IBD Medications and Drug Responses for external measurements. We were able to make use of the answers of 1961 out of 5355 patients (37%).

### Classification of disease subtypes

**Fig. 2:**
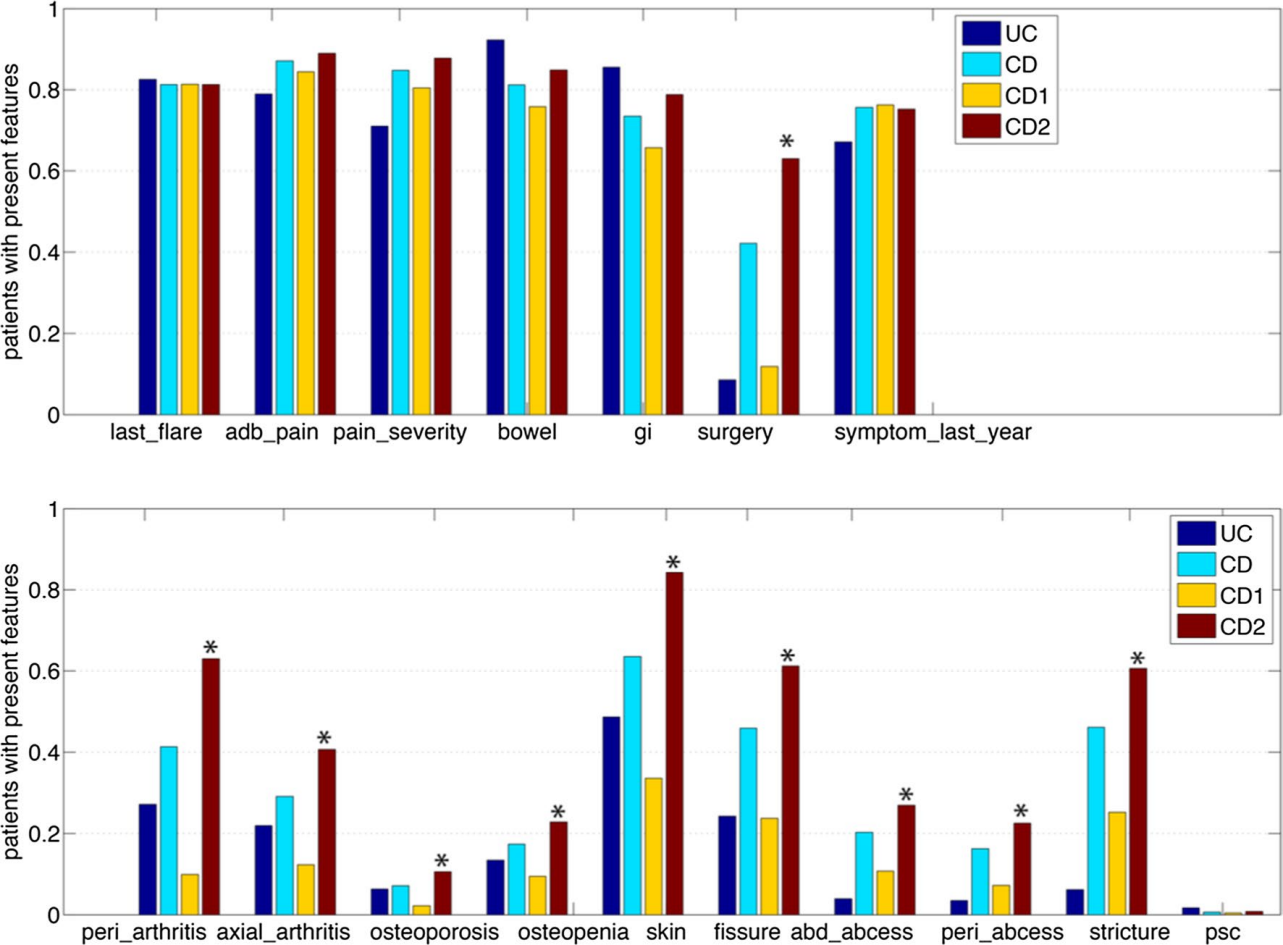
Compare feature enrichments in UC (843 patients), CD (1118 patients) and two CD subtypes: UC-like CD (456 CD1) and CD-like CD (662 CD2). The percentage of patients with present features in the subpopulation are plotted. Ten features that are significantly enriched in CD2 are marked with asterisks

The 1961 patients were clustered into two groups based on the seventeen phenotypic features using model-based clustering [23, 24]. Cluster-1 contains 791 UC and 456 CD patients, while Cluster-2 contains 52 UC and 662 CD patients. We compared the features of the 456 CD labeled patients in Cluster-1, in which UC patients dominate, and compared them to the 662 CD labeled patients from Cluster-2. Figure 2 shows enriched phenotypes in the two CD subtypes. The phenotypes are significantly different (Table 1): surgery_any, peripheral_arthritis, axial_arthritis, osteoporosis, osteopenia, skin, fissure, abdominal_abcess, perianal_abcess and stricture. The meanings of these phenotype terms are explained in Table 2. Not surprisingly, the features reported by patients in Cluster 2 labeled CD (Cluster-2 is made almost exclusively of CD labeled patients) are similar to the features that distinguish CD patients from UC patients more generally. Because UC segregates primarily into the first cluster, the interesting split is in the CD cases—which are almost evenly represented in the two clusters. Thus, it appears that the patient-reported data supports the clinical diagnosis of “UC” as a relatively homogenous phenotype, but also suggests that there is substantial heterogeneity in the CD patients, who we will refer to as “UC-like CD” (for CD labeled patients in Cluster-1) and “CD-like CD” (for CD labeled patients in Cluster-2).

**Table 1:** Compare phenotypic feature enrichments in UC-like CD (CD1) and CD-like CD (CD2)

Feature	Number of patients with the feature			Significance
	CD1(456)	CD2(662)	CD (1118)	− Log10(P)
last_flare	371 (81%)	538 (81%)	909 (81%)	1.206
typical_adbom_pain	385 (84%)	589 (89%)	974 (87%)	2.209
abdom_pain_severity	367 (80%)	581 (88%)	948 (85%)	3.547
bowel_movements	346 (76%)	562 (85%)	908 (81%)	4.288
gi_symptoms	300 (66%)	522 (79%)	822 (74%)	6.339
surgery_any	54 (12%)	417 (63%)	471 (42%)	**70.120**
symptoms_last_year	348 (76%)	498 (75%)	846 (76%)	1.284
peripheral_arthritis	45 (10%)	417 (63%)	462 (41%)	**77.016**
axial_arthritis	56 (12%)	269 (41%)	325 (29%)	**25.864**
osteoporosis	10 (2%)	70 (11%)	80 (7%)	**8.0374**
osteopenia	43 (9%)	151 (23%)	194 (17%)	**8.9286**
skin	153 (33%)	558 (84%)	711 (64%)	**68.174**
fissure	108 (24%)	405 (61%)	513 (46%)	**35.695**
abdominal_abcess	49 (11%)	178 (27%)	227 (20%)	**11.216**
perianal_abcess	33 (7%)	149 (22%)	182 (16%)	**12.046**
stricture	115 (25%)	401 (61%)	516 (46%)	**31.716**
psc	2 (0.4%)	5 (0.7%)	7 (0.6%)	0.594

Bold indicates the signficance is measured by -log10(P). The cutoff is >10

The number of patients with the feature observed in CD subtypes are listed in column 2 to 4. The hypergeometric *p* values for phenotypic feature enrichments are in column 5. Of the seventeen phenotypes, the enrichments for ten are significant (bold fonts), including surgery_any, peripheral_arthritis, axial_arthritis, osteoporosis, osteopenia, skin, fissure, abdominal_abcess, perianal_abcess and stricture

**Table 2:** The seventeen phenotypic features observed by patients who had a flare in the last 12 months

*last_flare:* When was your last IBD flare period?
( ) Less than 3 months ago [1]
( ) 3–6 months ago [1]
( ) 6–12 months ago [0]
( ) More than a year ago [removed]
*typical_adbom_pain:* How often were you troubled by pain in your abdomen during a typical flare last year?
( ) All of the time [1]
( ) Most of the time [1]
( ) A good bit of the time [1]
( ) Some of the time [1]
( ) A little of the time [0]
( ) Hardly any of the time [0]
( ) None of the time [0]
*abdom_pain_severity:* How intense was your worst pain rated on a 0–10 scale, where 0 is 'no pain' and 10 is 'pain as bad as could be' during a typical flare in the last year?
( ) 0–4 [0]
( ) 5–10 [1]
*bowel_movements*: Think about the baseline number of daily bowel movements you had before you got sick. During a typical flare in the past year, how has your number of daily bowel movements compared to this baseline?
( ) more daily bowel movements than my baseline [1]
( ) the same number of daily bowel movements as my baseline [0]
( ) I have had fewer daily bowel movements than my baseline[0]
*gi_symptoms*: During a typical flare in the last year, what digestion-related symptoms have you experienced?
Count the number of symptoms chosen. > = 3 [1] otherwise [0]
[ ] Diarrhea; [ ] Ribbon-shaped stool; [ ] Blood tinged stool; [ ] Blood in the toilet bowl;
[ ] Bloating; [ ] Abdominal pain; [ ] Excessive flatulence; [ ] None of the above
*surgery_any:* Have you undergone any surgeries to treat your IBD?
( ) yes [1] and ( ) no [0]
*symptoms_last_year:* Which of the following best describes your IBD-related symptoms in the last year?
( ) Remission (no symptoms) [0]
( ) Mild symptoms (some symptoms but no need for steroids or immunosuppressive or biologic medication) [0]
( ) Periods of flares and remissions (flares requiring steroids or immunosuppressive medication) [1]
( ) Chronically active disease (continual experience of moderate to severe symptoms) [1]
*peripheral_arthritis:* Joint pain or stiffness in your arms and legs
( ) yes [1] and ( ) no [0]
*axial_arthritis:* Joint pain or stiffness in your back or spine
( ) yes [1] and ( ) no [0]
*osteoporosis:* Osteoporosis
( ) yes [1] and ( ) no [0]
*osteopenia:* Low bone density or bone loss (osteopenia)
( ) yes [1] and ( ) no [0]
*skin*: Have you experienced any skin conditions?
( ) yes [1] and ( ) no [0]
*fissure:* A fissure (#40)
( ) yes [1] and ( ) no [0]
*abdominal_abcess:* An abscess in your abdomen
( ) yes [1] and ( ) no [0]
*perianal_abcess*: Perianal abscess (an infection around the anus)
( ) yes [1] and ( ) no [0]
*stricture*: A stricture (narrowing or blockage of the bowel)
( ) yes [1] and ( ) no [0]
*psc:* Primary sclerosing cholangitis (PSC)
( ) yes [1] and ( ) no [0]

The phenotype name is in italic font. We assessed each characteristic and assigned values (marked in square brackets)

### Associations between external evidence, drug response and disease subtypes

We retained questions in the section of IBD Background and Drug Responses (Additional file 1: Table S1) as external measurements to evaluate our classifications. We first analyzed the associations between smoking and the two CD subtypes (Table 3). A significantly higher percentage of CD-like CD patients (CD2) smoke, compared to UC-like CD patients, who also report lower severity. We also asked if gender plays a role as a modifier of CD transmission patterns. A significantly higher percentage of CD-like CD patients show maternal inheritance, compared to UC-like CD patients.

**Table 3:** Enrichments of external measurements

A. Smoking effects: number of smokers in different CD subtypes. Compared to UC-like CD patients (CD1), a significantly higher percentage of CD-like CD patients (CD2) smoke				
CD subtypes	CD1 (456)	CD2 (662)	CD (1118)	−Log10(P)
Smokers	108 (31%)	**247 (60%)**	355 (32%)	**6.3905**
B. Genetic effects: Compared to UC-like CD patients (CD1), a significantly higher percentage of CD-like CD patients (CD2) patients show maternal transmission				
CD subtypes	CD1 (456)	CD2 (662)	CD (1118)	−Log10(P)
Mother side	41(9%)	**106 (16%)**	147 (13%)	**3.7423**
Father side	47(10%)	76 (11%)	123 (11%)	1.1897
Total	88(19%)	182 (27%)	270 (24%)	3.4192

Bold indicates the signficance is measured by -log10(P). The cutoff is >3

We analyzed the drug response observed in 1118 CD patients of the two CD subtypes (456 UC-like CD and 662 CD-like CD). Of the six groups of drugs we analyzed, corticosteroids and salicylates are most frequently used in 87% and 74% of the 1118 CD patients, respectively (Table 4). For all the six groups, the percentage of users in CD-like CD is higher than that in UC-like CD. However, for three groups of drugs (salicylates, adalimumab, infliximab), the percentage of positive responses (effective) in UC-like CD is higher than that in CD-like CD. We analyzed the significance of positive drug response observed in different CD subtypes (Table 5). At a significance level 0.05, UC-like CD patients show more positive response to adalimumab, infliximab and salicylates, compared with CD-like CD. At a significance level 0.1, patients in CD-like CD show more positive response to corticosteroids, compared with those in UC-like CD. Overall, the prescription percentages of all drugs for CD-like CD patients are higher than UC-like CD, while the positive responses rate of TNF-inhibitors (adalimumab and infliximab) for UC-like CD patients are higher than CD-like CD patients.

**Table 4:** Drug responses in CD subtypes: UC-like CD patients (CD1), CD-like CD patients(CD2), and all CD patients

	CD1 (456)		CD2 (662)		CD (1118)	
	Prescription	Effective:Ineffective	Prescription	Effective:Ineffective	Prescription	Effective:Ineffective
Salicylates	290 (63%)	133:157 = 0.85	537 (81%)	205:332 = 0.62	827 (74%)	338:489 = 0.69
Antibiotics	154 (34%)	86:66 = 1.30	397 (60%)	248:149 = 1.66	549 (49%)	334:215 = 1.55
Corticosteroids	367 (80%)	294:73 = 4.03	608 (92%)	513:95 = 5.40	975 (87%)	807:168 = 4.80
Immunosuppressants	101 (22%)	43:58 = 0.74	249 (38%)	116:133 = 0.87	350 (31%)	159:191 = 0.83
Adalimumab	148 (32%)	115:33 = 3.48	324 (49%)	216:108 = 2.00	472 (42%)	331:141 = 2.35
Infliximab	153 (34%)	127:26 = 4.88	392 (59%)	291:101 = 2.88	545 (49%)	418:127 = 3.29

A higher percentage of patients in CD1 gave positive responses (effective) to adalimumab, infliximab and salicylates, even though the prescription rates are lower in CD1 compared with CD2 and all CD patients. A higher percentage of patients in CD2 gave positive responses to corticosteroids, compared with those in CD1 and all CD patients

**Table 5:** Significance of differentiated drug responses in CD subtypes

Drug	*p* value	Odd ratio (CD1:CD2)	Confidence interval	
			2.5%	97.5%
Salicylates	0.0322	1.372	1.027	1.832
Antibiotics	0.2074	0.783	0.534	1.146
Corticosteroids	0.0897	0.745	0.533	1.047
Immunosuppressants	0.4940	0.850	0.531	1.353
Adalimumab	0.0135	1.742	1.119	2.764
Infliximab	0.0259	1.695	1.064	2.781

At a significance level 0.05, CD1 patients gave more positive response (effective) to adalimumab, infliximab and salicylates, compared with CD2. At a significance level 0.1, CD2 patients gave more positive response to corticosteroids, compared with those in CD1. The odd ration of positive response in CD1 compared with CD2 are in column 3

### Detection of genotyping signature for CD subtypes

In order to seek genetic confirmation of these two different subtypes, we performed two genome wide association analyses to explore genetic differences (1) between Cluster-1 (791 UC and 456 CD patients, representing UC and UC-like phenotypes) and Cluster-2 (52 UC and 662 CD patients, representing CD patients), and (2) between CD-like CD and UC-like CD subtypes. Comparing Cluster-1 and Cluster-2 yielded multiple regions of significance. The highest ranked SNPs included variants from within the gene *ASH1L*, *MYOF*, and intergenic regions between *MYLK* and *CCDC14*, and *ZFR* and *SUB1* (Table 6). ASH1L has been previously shown to suppress inflammation by decreasing IL6 and TNF production and has been shown to be significantly downregulated in UC [26]. Surprisingly, comparing the two CD subtypes (456 UC-like CD and 662 CD-like CD) yielded no statistically significant SNPs, though the highest rank SNP, located between *SLC1A4* and *CEP68*, came close to statistical significance (Table 6). Other SNPs that were the most different between the two subtypes were in *RAB1A*, *TWIST2*, and between *TGIF2LX* and *PABPC5*.

**Table 6:** SNPs ranked by *p* value when comparing CD phenotypes versus UC and UC-like phenotypes, and CD-like CD versus UC-like CD subclusters

CD phenotypes versus UC and UC-like phenotypes			CD-like CD (CD1) versus UC-like CD (CD2)		
rsID	Gene context	*p* value	rsID	Gene context	*p* value
rs12025843	[ASH1L]	1.04e−9	rs115747343	SLC1A4--[]--CEP68	1.14e−7
rs201503922	[ASH1L]	2.86e−9	rs7423913	TWIST2-[]---HDAC4	2.88e−7
rs77037075	[ASH1L]	2.86e−9	rs78553357	SLC1A4--[]-CEP68	4.97e−7
rs74280600	MYLK--[]--CCDC14	5.58e−9	rs75143863	[RAB1A]	4.97e−7
rs75506868	[MYOF]	7.54e−9	rs60432037	[TWIST2]	4.98e−7
rs4867113	ZFR---[]--SUB1	8.44e−9	rs73633541	TGIF2LX---[]---PABPC5	8.24e−7

Gene context: [GENE] denotes within a gene, and GENE1---[]---GENE2 denotes relative position of SNP, denoted by brackets, between GENE1 and GENE2

## Discussion

### Two CD subtypes derived from patient-reported survey data

Patients clinically labeled with “CD” are a heterogeneous group in both their subjective symptoms and their response to drugs. Based on patient-reported information, we identified two CD subtypes, with one subtype of CD that has characteristics of UC. Genetic investigations have challenged the idea that IBD can be divided into two main diseases: CD that can affect any part of the digestive tract and UC that affects only the colon [9, 13]. Lees et al. [20] found that disease location separated out IBD much more naturally into three main disease types, rather than just two. They showed that small-bowel CD was distinct from colonic CD. Because we do not have disease localization information, we could not test the association of our CD subtypes with Lees’ classification. In addition, evidence supporting CD subtype classifications can be found in other recent research. It is known that about 10–15% of patients with colitis have disease features that do not permit definitive classification as either UC or CD of the colon and are labeled as having indeterminate colitis [13]. Current serologic and genetic studies, as well as endoscopic and imaging studies lack sufficient positive predictive values to make a definite diagnosis of CD or UC [11]. Although some patients with indeterminate colitis eventually develop characteristic UC or CD, a subgroup are durably indeterminate [13]. While traditional classification measures may provide clinical sub-phenotypes based on age at diagnosis and disease location and behavior, our classification represents an alternative (and simple) classification of CD into subtypes based on patient report.

We have used seventeen features based on patient-reported information, including abdominal pain, bowel movement and digestion-related symptoms. The features that are most enriched in CD-like CD are related to arthritis, osteoporosis and skin issues. We observed that CD-like CD patients have more severe symptoms than those UC-like CD patients, acknowledging that the concept of disease severity is difficult to quantify [12, 27]. Typically, when classifying patients for clinical studies, phenotype is determined at a specific time point. Disease behavior is surveyed retrospectively, and patients are not always labeled with a specific level of disease severity. In this regard, our classifications of disease behavior can provide a measure of severity. However, our results are limited to patients who have had a flare in the previous 12 months.

In addition, our analysis of drug response suggests the correlation between subtypes and severity as drugs are often more effective for patients of less severe symptoms. Salicylates constitute the first line of treatment for induction and maintenance of remission in UC. Corticosteroids constitute the second line of therapy in patients who fail to respond to the maximal dose of salicylates. Anti-TNF drugs are often used in patients with moderate-to-severe IBD who do not tolerate or respond to conventional therapies. The differential drug response to salicylates and TNF-inhibitors (Table 5) are as expected because the disease severity of CD-like CDs is greater. However, a higher percentage of positive response in CD-like CD for corticosteroids may suggest that CD-like CD may modulate T-cell activation and the production of pro-inflammatory cytokines (to which corticosteroids responds well.).

We also found associations between disease subtypes and external features that were not used for classification, including “smoking” and “maternal inheritance”. Cigarette smoking is a confirmed risk factor involved in the pathogenesis of Crohn’s disease, but seems to be a disease aggravating rather than a disease-causing factor [28]. It has been linked to disease progression and unfavorable disease outcome across the world. In fact, smokers are more likely to develop fistulae [29]. For “maternal inheritance”, it is not a surprise that genetic factors are also associated with CD severity. Previous studies suggest a greater rate of mother-to-child transmission in CD patients [30], suggesting a potential female sex-specific epigenetic inheritance pattern for CD. The CD-like CD subtype that we identified based on patients’ own observations may be relevant to family-specific risk of severe CD. These associations suggest the reasonableness of CD subtypes, indicating the usefulness of patients’ own observation in subtype classification.

The SNPs that were the most different between the two subtypes were in *RAB1A* and *TWIST2*. *RAB1A* is an oncogene that has been implicated in colon cancer [31], oral cancers [32, 33], and lung cancers [34]. *RAB1A* has been shown to be dysregulated in histologically normal lung tissue in smokers [35]. The differences in RAB1A may be associated with the observation of smoking differentiation between two CD subtypes. *TWIST2* represses NF-κβ and downregulates cytokines, including TNFα and IL1β by binding to E boxes in their promoter region [36]. As such, differences in *TWIST2* may contribute to differential drug response to salicylates, corticosteroids and TNF-inhibitors between the two CD subtypes. It may be that larger numbers would be required to find the genetic signal differentiating the two CD subtypes.

### Issues in the analysis of complex, contingent surveys

The survey we used was originally developed to support genetic association studies (unpublished data). Our analysis was initially motivated by the goal of defining more homogeneous populations for the GWAS analysis.

Compared with commonly used IBD patients’ questionnaires (i.e. the NIDDK IBD Genetics Consortium), the questions in this questionnaire cover similar content, but seek more details regarding disease behavior and drug responses. Many of these detailed features cannot be used in phenotype classifications because the questions were relevant to only a small number of respondents. For example, follow up questions after a conditional question only asked for the subset of subjects who answer affirmatively, and so the pattern of missingness in the data is largely determined by previous respondent answers. We identified several features in future patient surveys that might improve future analyses that aim to identify novel disease features.

First, to enable novel discoveries, survey designers should consider collecting information that is relevant to, and can be reliably provided by, all or most participants. The specific IBD phenotypic features should be easy to define and easy for all or most patients to report, such as the severity of common symptoms. Second, in order to reduce feature dependency, conditional questions should be minimized and structured to enable discovery of novel disease features. Initially, we processed all the questions in the section of IBD Background Questions and IBD Diagnosis and Symptoms and PCA analysis (Additional file 1: Figure S1) shows that two major sets of feature dependency are actually caused by conditional questions. We processed the two conditional questions as described in "Methods" (Select phenotypes and patients from questionnaire).

The questionnaire was designed to elicit detailed information about patient symptoms and drug response. As such, it created many useful subgroups of patients who were similar along these dimensions. However, for the purposes of discovering novel disease features and association with genetic signals, we needed to recombine these groups into more coarse categories. These groupings, although they are not as refined, allowed us to identify useful phenotypic distinctions between patients and suggested potential genetic associations.

## Conclusions

Are patient-reported phenotypes the best strategy to understand CD heterogeneity? Even with the limitations of a questionnaire designed to measure known disease characteristics, we were able to define two interesting new subtypes of patients clinically labeled as “CD.” We found sensible co-varying features and weak genetic signals. This indicates that patient-reported phenotypes are indeed useful for identifying disease subgroups. We show preliminary evidence that the subgroups correspond to some known risk factors, but also suggest some new associations from our analysis. Careful assessment of patient phenotypes may lead to improved phenotypic clusters, and opportunities for more targeted diagnosis and therapy.

## Supplementary Information

**Additional file 1.** Supplementary materials for data process and analysis.

**Additional file 2.** The original questionnaire.

## Data Availability

We made the original and the processed questionnaire available in Additional file 1. Individual-level survey, genotype data, and the datasets generated using the survey data or genotype data are not publicly available due participant confidentiality, and in accordance with the IRB-approved protocol under which the study was conducted.
