# Supplementary material for: Distinct clinical phenotypes for Crohn’s disease derived from patient surveys
Source: BMC Gastroenterol. 2021 Apr 9;21:160. doi: 10.1186/s12876-021-01740-6 (PMC8034169; doi:10.1186/s12876-021-01740-6)
Supplement: Supplementary file 1 — Additional file 1. Supplementary materials for data process and analysis. [file 12876_2021_1740_MOESM1_ESM.docx]

**Supplementary Material Part I**

**Distinct clinical phenotypes for Crohn’s disease derived from patient surveys**

Tianyun Liu^1^, Lichy Han^2^, Mera Tilley^3^, Lovisa Afzelius^3^, Mateusz Maciejewski^3^, Scott Jelinsky^3^, Chao Tian^4^, Matthew McIntyre^4^, the 23andMe Research Team^4^, Nan Bing^3^, Kenneth Hung^3^, Russ B Altman^1^

1. Department of Bioengineering, Stanford University, Stanford, CA
2. Biomedical Informatics Training Program, Stanford University, Stanford, CA
3. Inflammation & Immunology, Pfizer Inc., Cambridge, MA
4. 23andMe Research Team, 23andMe Inc., Sunnyvale, CA

Address correspondence to: Shriram Room 209, MC: 4245, 443 Via Ortega Drive, Stanford, CA 94305-4145. Email: russ.altman@stanford.edu

**Table S1. Selected questions for initial analysis.**

| **Index** | **Question** | **Index** | **Question** |
| --- | --- | --- | --- |
| **IBD Background** | | **Drug Responses** | |
| 1 | base_ever_smoker | 41 | base_infliximab_curr_use |
| 2 | base_mother_v2 | 42 | base_infliximab_duration |
| 3 | base_father_v2 | 43 | base_infliximab_work |
| 4 | base_mothers_mother_v2 | 44 | base_infliximab_work_std |
| 5 | base_mothers_father_v2 | 45 | base_infliximab_why_stop |
| 6 | base_fathers_mother_v2 | 46 | base_infliximab_side_eff_yn |
| 7 | base_fathers_father_v2 | 47 | base_adalimumab_curr_use |
| 8 | base_brothers_v2 | 48 | base_adalimumab_duration |
| 9 | base_sisters_v2 | 49 | base_adalimumab_work |
| 10 | base_children_v2 | 50 | base_adalimumab_work_std |
| **IBD Diagnosis and Symptom** | | 51 | base_adalimumab_why_stop |
| 11 | base_dx | 52 | base_adalimumab_side_eff_yn |
| **12** | **base_last_flare** | 53 | base_salicylates_curr_use |
| **13** | **base_typical_adbom_pain** | 54 | base_salicylates_duration |
| **14** | **base_abdom_pain_severity** | 55 | base_salicylates_work |
| **15** | **base_bowel_movements** | 56 | base_salicylates_work_std |
| **16** | **base_gi_symptoms** | 57 | base_salicylates_why_stop |
| **17** | **base_surgery_any** | 58 | base_salicylates_side_eff_yn |
| **18** | **base_symptoms_year_v2** | 59 | base_corticosteroids_curr_use |
| **19** | **base_peripheral_arthritis** | 60 | base_corticosteroids_duration |
| **20** | **base_axial_arthritis** | 61 | base_corticosteroids_work |
| **21** | **base_osteoporosis** | 62 | base_corticosteroids_work_std |
| **22** | **base_osteopenia** | 63 | base_corticosteroids_why_stop |
| **23** | **base_skin_yn** | 64 | base_corticosteroids_side_eff_yn |
| 24 | base_erythema_nodosum | 65 | base_immunosuppressants_curr_use |
| 25 | base_pyoderma_gangrenosum | 66 | base_immunosuppressants_duration |
| 26 | base_psoriasis | 67 | base_immunosuppressants_work |
| 27 | base_canker_sores | 68 | base_immunosuppressants_work_std |
| 28 | base_eczema | 69 | base_immunosuppressants_why_stop |
| **29** | **base_fissure** | 70 | base_immunosuppressants_side_eff_yn |
| **30** | **base_abdominal_abcess** | 71 | base_antibiotics_curr_use |
| **31** | **base_perianal_abcess** | 72 | base_antibiotics_duration |
| **32** | **base_stricture** | 73 | base_antibiotics_work |
| **33** | **base_psc** | 74 | base_antibiotics_work_std |
| **IBD Medications** | | 75 | base_antibiotics_work_std |
| 34 | base_salicylates | 76 | base_antibiotics_side_eff_yn |
| 35 | base_corticosteroids |  |  |
| 36 | base_immunosuppressants |  |  |
| 37 | base_acute_immunosuppressants |  |  |
| 38 | base_tnf_inhibitor |  |  |
| 39 | base_antibiotics |  |  |
| 40 | base_other_meds_v2 |  |  |
| **IBD Background** | | **Drug Responses** | |

**Notes: detailed methods in question selection**

Of the 143 questions, fifteen questions are in the section of IBD Background Questions, thirty questions in the section of IBD Diagnosis and Symptoms, seven general questions about medication in the section of IBD Medications. In the section of Drug Responses, patients answered seven questions about drug response for each of the thirteen drug groups (salicylates, corticosteroids, antibiotics, two groups of immunosuppressants, nine groups of TNF-inhibitors), resulting in 91 questions.

For questions in the sections of IBD Background Questions and IBD Diagnosis and Symptoms, we removed the following: (1) We removed four questions that addresses the year of diagnosis and disease onset because we could not track down the age of diagnosis and the age of disease onset due to the lack of birthdate information. (2) We removed the question “In the past year, has your IBD caused any of the following symptoms elsewhere in your body? Please select all that apply”, because patients could select any combinations of the seven symptoms provided, which resulted in wide variety of answers. (3) We removed two questions regarding to fistula because only a very small population has (<5%) this issue. (4) We removed six questions about background because of high percentage of “NA” in their answers. This led to ten questions in IBD Background Questions and 23 questions in IBD Diagnosis and Symptoms. The seventeen questions used for phenotype classification are in bold font in Table S1.

We kept all seven questions in the section of IBD Medications. The answers to the question about drug combinations has wide variety. Originally there are nine groups of TNF-inhibitors in the section of Drug Responses, but we had to remove seven groups of TNF-inhibitors because patients’ answers contain a very high level of “NA” (98%). One explanation is that these seven groups of TNF-inhibitors are rarely prescribed to patients. The two groups of TNF-inhibitors are kept for this study are infliximab and adalimumab. Drugs other than TNF-inhibitors in this study include saliclyaltes, corticosteroids, immunosuppressants and antibiotics. For each of the drug group, originally there are seven questions. However, the answers for “How well did DRUG work for you {IF ibd_DRUG_curr_use=no}” and for “How well did DRUG work for you {IF ibd_DRUG_curr_use=YES}” are identical for all drugs. We therefor merged these two questions as one, with six questions left for each drug group. In summary, there are total of ten background questions (NA percentage: 4%-40%); 23 symptom questions (NA percentage: 5%-49%); seven general medication questions (NA percentage: 6%-7%); twelve questions for TNF-inhibitors (NA percentage:64%-90%), 24 questions for other drugs (20%-98%). These 76 questions are labeled with new index as shown in Table S1.

**Table S2.** Survey questions and answer options. The new index of selected questions in Table S1 are marked in squared brackets at the end of the question. The questions not included in Table S1 marked with [D].

**[1] PAGE: IBD Background Questions**
1. What is your sex? [D]

2. {IF sex = female} Have you ever used birth control pills? [D]
3. Have you ever smoked cigarettes on a regular basis? [1]

4. {IF ever smoked} Do you currently smoke cigarettes? [D]
5.  {IF currently smoking} How many cigarettes do you smoke on a typical day? [D]
6.  {IF currently smoking} How old were you when you first started to smoke regularly? [D]

Have any of the following biological relatives been diagnosed with Crohn's disease or ulcerative colitis?

7. Your mother [2]

8. Your father [3]

9. Your mother's mother [4]

10. Your mother's father [5]

11. Your father's mother [6]

12. Your father's father [7]

13. Your brothers [8]

14. Your sisters [9]

15. Your children [10]

**[2] PAGE: IBD Diagnosis and Symptoms**

16. What kind of IBD do you have? [11]

17. {IF diagnosed with Crohn’s} In what year did a doctor first diagnose you with Crohn's disease? [D]

18. {IF diagnosed with Crohn’s} In what year did you first start experiencing symptoms of Crohn's disease? [D]

19. {IF diagnosed with UC} In what year did a doctor first diagnose you with ulcerative colitis? [D]

20. {IF diagnosed with UC} In what year did you first start experiencing symptoms of ulcerative colitis? [D]
21. When was your last IBD flare period? [12]

22. {IF had a flare in the last 12 months} How often were you troubled by pain in your abdomen *during a typical flare* in the last year? [13]   
23. {IF had a flare in the last 12 months} How intense was your worst pain rated on a 0-10 scale, where 0 is 'no pain' and 10 is 'pain as bad as could be' *during a typical flare* in the last year?    {IF had a flare in the last 12 months} [14]
24. {IF had a flare in the last 12 months} Think about the baseline number of daily bowel movements you had before you got sick. *During a typical flare* in the past year, how has your number of daily bowel movements compared to this baseline? [15]
25. {IF had a flare in the last 12 months} *During a typical flare* in the last year, what digestion-related symptoms have you experienced? [16]
26. *In the past year*, has your IBD caused any of the following symptoms elsewhere in your body? Please select all that apply. [D]

27. Have you undergone any surgeries to treat your IBD? [17]
28. Which of the following best describes your IBD-related symptoms in the last year?[18]

Has a doctor diagnosed you with any of the following joint or bone conditions?

29. Joint pain or stiffness in your arms and legs, including the elbows, wrists, knees, and ankles [19]

30. Joint pain or stiffness in your back or spine [20]

31. Osteoporosis [21]

32. Low bone density or bone loss (osteopenia) [22]

33. Have you experienced any skin conditions or mouth sores since being diagnosed with IBD? [23]

{IF ibd_skin_yn = yes} Which skin conditions have you experienced?

34. Erythema nodosum (tender red nodules on the shins and legs) [24]

35. Pyoderma gangrenosum (deep ulcers of the skin that heal poorly) [25]

36. Psoriasis (dry red patches of skin covered with scales) [26]

37. Small ulcers in the mouth (aphthous stomatitis or "canker sores.") [27]

38. Eczema [28]

Has a doctor diagnosed you with any of the following problems?

39. A fistula (an abnormal tunnel connecting two body cavities or a body cavity and the skin) [D]

40. A fissure (tears or areas of tender redness around rectal areas) [29]

41. An abscess in your abdomen (an infection inside the abdomen) [30]

42. Perianal abscess (an infection around the anus) [31]

43. A stricture (narrowing or blockage of the bowel) [32]

44. Primary sclerosing cholangitis (PSC) [33]

45. {IF had a fistula} What kind of fistula did you have? [D]

**Table S3.** The significant difference between UC-like CD patients (CD1) and CD-like CD patients (CD2) are reflected in bowel_movements, gi_symptoms, surgery_any, peripheral_arthritis, axial_arthritis, osteoporosis, osteopenia, skin, fissure, abdominal_abcess, perianal_abcess*,* and stricture*.*

| **phenotype** | **UC (843)** | **CD1(456)** | **CD2(662)** | **CD (1118)** |
| --- | --- | --- | --- | --- |
| last_flare | 0.8256 | 0.8136 | 0.8127 | 0.8131 |
| typical_adbom_pain | 0.7900 | 0.8443 | 0.8897 | 0.8712 |
| abdom_pain_severity | 0.7106 | 0.8048 | 0.8776 | 0.8479 |
| bowel_movements | 0.9229 | 0.7588 | 0.8489 | 0.8122 |
| gi_symptoms | 0.8553 | 0.6579 | 0.7885 | 0.7352 |
| surgery_any | 0.0854 | 0.1184 | 0.6229 | 0.4213 |
| symptoms_last_year | 0.6714 | 0.7632 | 0.7523 | 0.7567 |
| peripheral_arthritis | 0.2716 | 0.0987 | 0.6443 | 0.4132 |
| axial_arthritis | 0.2195 | 0.1228 | 0.4063 | 0.2907 |
| osteoporosis | 0.0629 | 0.0219 | 0.1057 | 0.0716 |
| osteopenia | 0.1340 | 0.0943 | 0.2281 | 0.1735 |
| skin | 0.4864 | 0.3355 | 0.8429 | 0.6360 |
| fissure | 0.2420 | 0.2368 | 0.6118 | 0.4589 |
| abdominal_abcess | 0.0391 | 0.1075 | 0.2689 | 0.2030 |
| perianal_abcess | 0.0344 | 0.0724 | 0.2251 | 0.1628 |
| stricture | 0.0617 | 0.2522 | 0.6057 | 0.4615 |
| psc | 0.0166 | 0.0044 | 0.0076 | 0.0063 |

**Table S4**. External measurements characterize two CD types.

1. Smoking is enriched in CD2 patients.

| **External evidence** | **UC (843)** | **CD1(456)** | **CD2(662)** | **CD (1118)** |
| --- | --- | --- | --- | --- |
| smoke | 262/574=0.45 | 108/346=0.31 | 247/409=0.60 | 355/755=0.47 |

1. Parent transmission ratio is higher in CD2 patients.

| **External evidence** | **UC (843)** | **CD1(456)** | **CD2(662)** | **CD (1118)** |
| --- | --- | --- | --- | --- |
| mother | 61 | 30 | 67 | 97 |
| father | 41 | 23 | 43 | 66 |
| mother/mother | 33 | 7 | 24 | 31 |
| mother/father | 18 | 4 | 15 | 19 |
| father/mother | 31 | 13 | 24 | 37 |
| father/father | 12 | 11 | 9 | 20 |
| Mother side | 112 | 41 | 106 | 147 |
| Father side | 84 | 47 | 76 | 123 |
| total | 196 | 88 | 182 | 270 |
| ratio | 0.23 | 0.19 | 0.28 | 0.24 |

**Table S5.** Positive drug responses ratio in UC, CD and two CD types.

| **Drug** | **UC (843)** | **CD1(456)** | **CD2(662)** | **CD (1118)** |
| --- | --- | --- | --- | --- |
| salicylates | 450:273=1.65 | 133:157=0.85 | 205:332=0.62 | 338:489=0.69 |
| antibiotics | 139:116=1.20 | 86:66=1.30 | 248:149=1.66 | 334:215=1.55 |
| corticosteroids | 563:119=4.73 | 294:73=4.03 | 513:95=5.40 | 807:168=4.80 |
| immunosuppressants | 73:83=0.88 | 43:58=0.74 | 116:133=0.87 | 159:191=0.83 |
| adalimumab | 78:61=1.28 | 115:33=3.48 | 216:108=2.00 | 331:141=2.35 |
| infliximab | 132:59=2.24 | 127:26=4.88 | 291:101=2.88 | 418:127=3.29 |

**Figure S1**. Feature dependency caused by questionnaire design. We analyzed the dependency between the 33 questions in the section of IBD Background Questions and IBD Diagnosis and Symptoms. PCA analysis shows that the dependency of some features can be caused by two conditional questions.

(1). In the section of IBD Diagnosis and Symptoms, when answering to the question (#12) “When was your last IBD flare period?”, the answer options are: “Less than 3 months ago”, “3 – 6 months ago”, “6 – 12 months ago” and “More than a year ago”. According to answers to question #12, patients were divided into two groups: those who had a flare in the last twelve months and those who had not*.* Patients who did not have flare in the last twelve months did not provide answers to the next four questions regarding to the symptoms during a flare, resulting in “NA” as their answers to those questions. (#13 How often were you troubled by pain in your abdomen during a typical flare in the last year? #14 How intense was your worst pain rated on a 0-10 scale during a flare? #15 During a typical flare in the past year, how has your number of daily bowel movements compared to this baseline? #16 During a typical flare in the last year, what digestion-related symptoms have you experienced?). This is shown in the bottom box.

(2). If a patient’s answer to question #23 “Have you experienced any skin conditions or mouth sores since being diagnosed with IBD?” is “YES”, the patient is asked to answer a series of questions “Which skin conditions have you experienced?”(#24 to # 28: Erythema nodosum, Pyoderma gangrenosum, Psoriasis, Small ulcers in the mouth, Eczema). Otherwise, patients can skip questions #24 to #28, leaving a very large number of “NA” in these four features. This is shown in the box on the very right side.
