# Supplementary material for: Distinct clinical phenotypes for Crohn’s disease derived from patient surveys
Source: BMC Gastroenterol. 2021 Apr 9;21:160. doi: 10.1186/s12876-021-01740-6 (PMC8034169; doi:10.1186/s12876-021-01740-6)
Supplement: Supplementary file 2 — Additional file 2. The original questionnaire. [file 12876_2021_1740_MOESM2_ESM.docx]

**Supplementary Material Part II**

**Distinct clinical phenotypes for Crohn’s disease derived from patient surveys**

Tianyun Liu^1^, Lichy Han^2^, Mera Tilley^3^, Lovisa Afzelius^3^, Mateusz Maciejewski^3^, Scott Jelinsky^3^, Chao Tian^4^, Matthew McIntyre^4^, the 23andMe Research Team^4^, Nan Bing^3^, Kenneth Hung^3^, Russ B Altman^1^

1. Department of Bioengineering, Stanford University, Stanford, CA
2. Biomedical Informatics Training Program, Stanford University, Stanford, CA
3. Inflammation & Immunology, Pfizer Inc., Cambridge, MA
4. 23andMe Research Team, 23andMe Inc., Sunnyvale, CA

Address correspondence to: Shriram Room 209, MC: 4245, 443 Via Ortega Drive, Stanford, CA 94305-4145. Email: russ.altman@stanford.edu

**SURVEY TITLE:** Inflammatory Bowel Disease Community Survey
**SURVEY DESCRIPTION:** Thank you for taking this survey for our Inflammatory Bowel Disease (IBD) research study! We will use your answers to this and other surveys to investigate how genetic differences affect the causes and severity of IBD, and how well IBD symptoms respond to treatments.

Estimated time to complete: 15-25 minutes

The first page asks about aspects of your general health and family history.

**[1] PAGE: IBD Background Questions**

1. What is your sex?
    ( ) Male
    ( ) Female
    ( ) Other

    {IF sex = female}
2. Have you ever used birth control pills?
    ( ) Yes
    ( ) No
    ( ) I'd rather not say

3. Have you ever smoked cigarettes on a regular basis?
    ( ) Yes
    ( ) No
    ( ) I'd rather not say

    {IF ever smoked}
4. Do you currently smoke cigarettes?
    ( ) Yes
    ( ) No
    ( ) I'd rather not say

    {IF currently smoking}
5. How many cigarettes do you smoke on a typical day?
    ( ) Cigarettes per day:
    ( ) Packs per day:

    {IF currently smoking}
6. How old were you when you first started to smoke regularly?

____ [Integer between 1 and 99]

Have any of the following biological relatives been diagnosed with Crohn's disease or ulcerative colitis?
7. Your mother

( )Yes    ( )No    ( )Not applicable

8. Your father

( )Yes    ( )No    ( )Not applicable

9. Your mother's mother

( )Yes    ( )No    ( )Not applicable

10. Your mother's father

( )Yes    ( )No    ( )Not applicable

11. Your father's mother

( )Yes    ( )No    ( )Not applicable

12. Your father's father

( )Yes    ( )No    ( )Not applicable

13. Your parent's siblings

( )Yes    ( )No    ( )Not applicable

14. Your siblings

( )Yes    ( )No    ( )Not applicable

15. Your children

( )Yes    ( )No    ( )Not applicable

16. What kind of IBD do you have?
    ( ) Crohn's disease
    ( ) Ulcerative colitis
    ( ) I have another type of inflammatory bowel disease
    ( ) I do not have inflammatory bowel disease

    {IF diagnosed with Crohn’s or UC}
**[2] PAGE: IBD Diagnosis and Symptoms**

    {IF diagnosed with Crohn’s}
17. In what year (YYYY) did a doctor first diagnose you with Crohn's disease?

    {IF diagnosed with Crohn’s}
18. In what year (YYYY) did you first start experiencing symptoms of Crohn's disease?

    {IF diagnosed with UC}
19. In what year (YYYY) did a doctor first diagnose you with ulcerative colitis?

    {IF diagnosed with UC}
20. In what year (YYYY) did you first start experiencing symptoms of ulcerative colitis?

21. When was your last IBD flare period?
    ( ) Less than 3 months ago
    ( ) 3 - 6 months ago
    ( ) 6 - 12 months ago
    ( ) More than a year ago

    {IF had a flare in the last 12 months}

22. How often were you troubled by pain in your abdomen *during a typical flare* in the last year?
    ( ) All of the time
    ( ) Most of the time
    ( ) A good bit of the time
    ( ) Some of the time
    ( ) A little of the time
    ( ) Hardly any of the time
    ( ) None of the time

    {IF had a flare in the last 12 months}
23. How intense was your worst pain rated on a 0-10 scale, where 0 is 'no pain' and 10 is 'pain as bad as could be' *during a typical flare* in the last year?
    ( ) 0 (No pain)
    ( ) 1
    ( ) 2
    ( ) 3
    ( ) 4
    ( ) 5
    ( ) 6
    ( ) 7
    ( ) 8
    ( ) 9
    ( ) 10 (Pain as bad as it could be)

    {IF had a flare in the last 12 months}
24. Think about the baseline number of daily bowel movements you had before you got sick. *During a typical flare* in the past year, how has your number of daily bowel movements compared to this baseline?
    ( ) I have had more daily bowel movements than my baseline
    ( ) I have had the same number of daily bowel movements as my baseline
    ( ) I have had fewer daily bowel movements than my baseline

    {IF had a flare in the last 12 months}
25. *During a typical flare* in the last year, what digestion-related symptoms have you experienced?
    [ ] Diarrhea
    [ ] Ribbon-shaped stool
    [ ] Blood tinged stool
    [ ] Blood in the toilet bowl
    [ ] Bloating
    [ ] Abdominal pain
    [ ] Excessive flatulence
    [ ] None of the above

26. *In the past year*, has your IBD caused any of the following symptoms elsewhere in your body? Please select all that apply.
    [ ] Pain in hands
    [ ] Pain in joints or swollen joints
    [ ] Skin rash
    [ ] Eye pain or redness (Episcleritis, Iritis, Uveitis)
    [ ] Anemia (low blood iron levels)
    [ ] Blot clots and/or pulmonary embolism (venous thromboembolism)
    [ ] None of the above

27. Have you undergone any surgeries to treat your IBD?
    ( ) Yes
    ( ) No

28. Which of the following best describes your IBD-related symptoms in the last year?
    ( ) Remission (no symptoms)
    ( ) Mild symptoms (some symptoms but no need for steroids or immunosuppressive or biologic medication)
    ( ) Periods of flares and remissions (flares requiring steroids or immunosuppressive medication)
    ( ) Chronically active disease (continual experience of moderate to severe symptoms)

Has a doctor diagnosed you with any of the following joint or bone conditions?
29. Joint pain or stiffness in your arms and legs, including the elbows, wrists, knees, and ankles

( )Yes    ( )No    ( )I'm not sure

30. Joint pain or stiffness in your back or spine

( )Yes    ( )No    ( )I'm not sure

31. Osteoporosis

( )Yes    ( )No    ( )I'm not sure

32. Low bone density or bone loss (osteopenia)

( )Yes    ( )No    ( )I'm not sure

33. Have you experienced any skin conditions or mouth sores since being diagnosed with IBD?
    ( ) Yes
    ( ) No

    {IF ibd_skin_yn = yes}Which skin conditions have you experienced?
34. Erythema nodosum (tender red nodules on the shins and legs)

( )Yes    ( )No    ( )I'm not sure

35. Pyoderma gangrenosum (deep ulcers of the skin that heal poorly)

( )Yes    ( )No    ( )I'm not sure

36. Psoriasis (dry red patches of skin covered with scales)

( )Yes    ( )No    ( )I'm not sure

37. Small ulcers in the mouth (aphthous stomatitis or "canker sores.")

( )Yes    ( )No    ( )I'm not sure

38. Eczema

( )Yes    ( )No    ( )I'm not sure

Has a doctor diagnosed you with any of the following problems?
39. A fistula (an abnormal tunnel connecting two body cavities or a body cavity and the skin)

( )Yes    ( )No    ( )I'm not sure

40. A fissure (tears or areas of tender redness around rectal areas)

( )Yes    ( )No    ( )I'm not sure

41. An abscess in your abdomen (an infection inside the abdomen)

( )Yes    ( )No    ( )I'm not sure

42. Perianal abscess (an infection around the anus)

( )Yes    ( )No    ( )I'm not sure

43. A stricture (narrowing or blockage of the bowel)

( )Yes    ( )No    ( )I'm not sure

44. Primary sclerosing cholangitis (PSC)

( )Yes    ( )No    ( )I'm not sure

    {IF had a fistula}
45. What kind of fistula did you have?
    ( ) Enterocutaneous (a fistula from the intestine to the abdominal wall, for example, from your intestine to your belly button)
    ( ) Perianal (a fistula from your rectum to your buttocks)
    ( ) Enteroenteric or Enterocolic (a fistula that involves the large or small intestine)
    ( ) Enterovaginal (a fistula that goes to the vagina)
    ( ) Enterovesicular (a fistula that goes to the bladder)

    {IF diagnosed with Crohn’s or UC}
**[3] PAGE: IBD Medications**

The following questions ask about medications you have **ever taken** to treat your inflammatory bowel disease (IBD). For each medication type, both brand and generic names are listed. 

Please provide as much information as you can. If you don't recognize any of the medications listed, please select 'None of the above'.

46. The following medications reduce inflammation in similar ways and are called **salicylates**. Have you ever taken any of them to treat your IBD and related symptoms? Please check all that apply.
    [ ] 5-aminosalicylic acid (5-ASA, mesalamine, mesalazine, Apriso, Asacol, Canasa, Lialda, Mesasal, Mezavant, Pentasa, Rowasa, Salofalk)
    [ ] Balsalazide (Colazal, Colazide, MMX)
    [ ] Olsalazine (Dipentum)
    [ ] Sulfasalazine (Azulfidine, Salazopyrin, Sulazine)
    [ ] I'm not sure
    [ ] None of the above

47. The following medications reduce inflammation in similar ways and are called **corticosteroids**. Have you ever taken any of them to treat your IBD and related symptoms? Please check all that apply.
    [ ] Prednisone (prednisolone, Deltasone, Winpred)
    [ ] Entocort
    [ ] Methylprednisolone (Medrol, Meprolone, SoluMedrol)
    [ ] Hydrocortisone (Colocort, Cortef, Cortenema, Cortifoam, Cortisol, SoluCortef)
    [ ] Corticotropin (ACTH, H.P. Acthar)
    [ ] Betamethasone (Betnesol, Betnovate, Celestone)
    [ ] Uceris
    [ ] Dexamethasone (dexamethasone acetate, dexamethasone intensol, Dexacen LA-8, Dexasone, Dexpak)
    [ ] Other, please specify:_____________
    [ ] I'm not sure
    [ ] None of the above

48. The following medications suppress the immune system in similar ways. Have you ever taken any of them to treat your IBD and related symptoms? Please check all that apply.
    [ ] Azathioprine (AZA, Azasan, Imuran)
    [ ] 6-Mercaptopurine (6-MP, mercaptopurine, Purinethol)
    [ ] Methotrexate (MTX, Rheumatrex)
    [ ] I'm not sure
    [ ] None of the above

49. The following medications are used over brief periods to suppress very severe autoimmune reactions. Have you ever taken either of them? Please check all that apply.
    [ ] Tacrolimus (Advagraf, Prograf)
    [ ] Ciclosporin (cyclosporine, cyclosporin, cyclosporine A, csA, Neoral, Sandimmune)
    [ ] I'm not sure
    [ ] None of the above

50. The following medications reduce inflammation in similar ways and are called **TNF inhibitors**. Have you ever taken any of them to treat your IBD and related symptoms? Please check all that apply.
    [ ] Adalimumab (Humira)
    [ ] Certolizumab pegol (Cimzia)
    [ ] Infliximab (Remicade)
    [ ] Golimumab (Simponi)
    [ ] I'm not sure
    [ ] None of the above

51. The following medications are antibiotics that are sometimes used to treat IBD. Have you ever taken any of them to treat your IBD and related symptoms? Please check all that apply.
    [ ] Metronidazole
    [ ] Ciprofloxacin
    [ ] Rifaximin
    [ ] Clarithromycin
    [ ] I'm not sure
    [ ] None of the above

52. Have you ever taken either of these two medications to treat your IBD and related symptoms? Please check all that apply.
    [ ] Natalizumab (Tysabri)
    [ ] Ustekinumab (Stelara)
    [ ] Vedolizumab (Entyvio)
    [ ] I'm not sure
    [ ] None of the above

    {IF diagnosed with Crohn’s or UC, AND ever took tacrolimus}
**[4] PAGE: Advagraf, Prograf, or tacrolimus**

This page asks about your use of Advagraf, Prograf or tacrolimus to control your IBD symptoms.

53. Are you currently taking Advagraf®, Prograf®, or tacrolimus?
    ( ) Yes
    ( ) No

    {IF ibd_tacrolimus_curr_use = yes}
54. What is the longest period of time you took Advagraf®, Prograf®, or tacrolimus without any interruption of more than 2 months?
    ( ) Less than 4 months
    ( ) 4 months - 1 year
    ( ) 1 - 5 years
    ( ) Over 5 years

    {IF ibd_tacrolimus_curr_use = yes}
55. After using Advagraf®, Prograf®, or tacrolimus for several months, how were your symptoms compared to your baseline?
    ( ) Symptoms got much worse
    ( ) Symptoms got a little worse
    ( ) Symptoms neither worsened nor improved
    ( ) Symptoms improved a little
    ( ) Symptoms improved a lot

    {IF ibd_tacrolimus_curr_use = no}
56. How well did Advagraf®, Prograf®, or tacrolimus work for you?
    ( ) Not at all
    ( ) A little
    ( ) Somewhat
    ( ) A fair amount
    ( ) A great deal

    {IF ibd_tacrolimus_curr_use = yes}
57. How well does Advagraf®, Prograf®, or tacrolimus work for you?
    ( ) Not at all
    ( ) A little
    ( ) Somewhat
    ( ) A fair amount
    ( ) A great deal

    {IF ibd_tacrolimus_curr_use = no}
58. Why did you stop taking Advagraf®, Prograf®, or tacrolimus?
    [ ] Side effects
    [ ] The medication did not control my IBD symptoms enough
    [ ] Cost or insurance coverage
    [ ] Other

    {IF ibd_tacrolimus_curr_use = no}
59. Did you have any side effects while taking Advagraf®, Prograf®, or tacrolimus?
    ( ) Yes
    ( ) No

    {IF diagnosed with Crohn’s or UC, AND ever took cyclosporin}
 **[5] PAGE: cyclosporine, cyclosporin, cyclosporine A, csA, Neoral, Sandimmune or ciclosporin**

This page asks about your use of cyclosporine, cyclosporin, cyclosporine A, csA, Neoral, Sandimmune or ciclosporin to control your IBD symptoms.

60. Are you currently taking cyclosporine®, cyclosporin®, cyclosporine A®, csA®, Neoral®, Sandimmune® or ciclosporin?
    ( ) Yes
    ( ) No

    {IF ibd_ciclosporin_curr_use = yes}
61. What is the longest period of time you took cyclosporine®, cyclosporin®, cyclosporine A®, csA®, Neoral®, Sandimmune® or ciclosporin without any interruption of more than 2 months?
    ( ) Less than 4 months
    ( ) 4 months - 1 year
    ( ) 1 - 5 years
    ( ) Over 5 years

    {IF ibd_ciclosporin_curr_use = yes}
62. After using cyclosporine®, cyclosporin®, cyclosporine A®, csA®, Neoral®, Sandimmune® or ciclosporin for several months, how were your symptoms compared to your baseline?
    ( ) Symptoms got much worse
    ( ) Symptoms got a little worse
    ( ) Symptoms neither worsened nor improved
    ( ) Symptoms improved a little
    ( ) Symptoms improved a lot

    {IF ibd_ciclosporin_curr_use = no}
63. How well did cyclosporine®, cyclosporin®, cyclosporine A®, csA®, Neoral®, Sandimmune® or ciclosporin work for you?
    ( ) Not at all
    ( ) A little
    ( ) Somewhat
    ( ) A fair amount
    ( ) A great deal

    {IF ibd_ciclosporin_curr_use = yes}
64. How well does cyclosporine®, cyclosporin®, cyclosporine A®, csA®, Neoral®, Sandimmune® or ciclosporin work for you?
    ( ) Not at all
    ( ) A little
    ( ) Somewhat
    ( ) A fair amount
    ( ) A great deal

    {IF ibd_ciclosporin_curr_use = no}
65. Why did you stop taking cyclosporine®, cyclosporin®, cyclosporine A®, csA®, Neoral®, Sandimmune® or ciclosporin?
    [ ] Side effects
    [ ] The medication did not control my IBD symptoms enough
    [ ] Cost or insurance coverage
    [ ] Other

    {IF ibd_ciclosporin_curr_use = no}
66. Did you have any side effects while taking cyclosporine®, cyclosporin®, cyclosporine A®, csA®, Neoral®, Sandimmune® or ciclosporin?
    ( ) Yes
    ( ) No

    {IF diagnosed with Crohn’s or UC, AND ever took infliximab}
**[6] PAGE: Remicade or infliximab**

This page asks about your use of Remicade or infliximab to control your IBD symptoms.

67. Are you currently taking Remicade® or infliximab?
    ( ) Yes
    ( ) No

    {IF ibd_infliximab_curr_use = yes}
68. What is the longest period of time you took Remicade® or infliximab without any interruption of more than 2 months?
    ( ) Less than 4 months
    ( ) 4 months - 1 year
    ( ) 1 - 5 years
    ( ) Over 5 years

    {IF ibd_infliximab_curr_use = yes}
69. After using Remicade® or infliximab for several months, how were your symptoms compared to your baseline?
    ( ) Symptoms got much worse
    ( ) Symptoms got a little worse
    ( ) Symptoms neither worsened nor improved
    ( ) Symptoms improved a little
    ( ) Symptoms improved a lot

    {IF ibd_infliximab_curr_use = no}
70. How well did Remicade® or infliximab work for you?
    ( ) Not at all
    ( ) A little
    ( ) Somewhat
    ( ) A fair amount
    ( ) A great deal

    {IF ibd_infliximab_curr_use = yes}
71. How well does Remicade® or infliximab work for you?
    ( ) Not at all
    ( ) A little
    ( ) Somewhat
    ( ) A fair amount
    ( ) A great deal

    {IF ibd_infliximab_curr_use = no}
72. Why did you stop taking Remicade® or infliximab?
    [ ] Side effects
    [ ] The medication did not control my IBD symptoms enough
    [ ] Cost or insurance coverage
    [ ] Other

    {IF ibd_infliximab_curr_use = no}
73. Did you have any side effects while taking Remicade® or infliximab?
    ( ) Yes
    ( ) No

    {IF diagnosed with Crohn’s or UC, AND ever took adalimumab}
**[7] PAGE: HUMIRA or adalimumab**

This page asks about your use of HUMIRA or adalimumab to control your IBD symptoms.

74. Are you currently taking HUMIRA® or adalimumab?
    ( ) Yes
    ( ) No

    {IF ibd_adalimumab_curr_use = yes}
75. What is the longest period of time you took HUMIRA® or adalimumab without any interruption of more than 2 months?
    ( ) Less than 4 months
    ( ) 4 months - 1 year
    ( ) 1 - 5 years
    ( ) Over 5 years

    {IF ibd_adalimumab_curr_use = yes}
76. After using HUMIRA® or adalimumab for several months, how were your symptoms compared to your baseline?
    ( ) Symptoms got much worse
    ( ) Symptoms got a little worse
    ( ) Symptoms neither worsened nor improved
    ( ) Symptoms improved a little
    ( ) Symptoms improved a lot

    {IF ibd_adalimumab_curr_use = no}
77. How well did HUMIRA® or adalimumab work for you?
    ( ) Not at all
    ( ) A little
    ( ) Somewhat
    ( ) A fair amount
    ( ) A great deal

    {IF ibd_adalimumab_curr_use = yes}
78. How well does HUMIRA® or adalimumab work for you?
    ( ) Not at all
    ( ) A little
    ( ) Somewhat
    ( ) A fair amount
    ( ) A great deal

    {IF ibd_adalimumab_curr_use = no}
79. Why did you stop taking HUMIRA® or adalimumab?
    [ ] Side effects
    [ ] The medication did not control my IBD symptoms enough
    [ ] Cost or insurance coverage
    [ ] Other

    {IF ibd_adalimumab_curr_use = no}
80. Did you have any side effects while taking HUMIRA® or adalimumab?
    ( ) Yes
    ( ) No

    {IF diagnosed with Crohn’s or UC, AND ever took certolizumab}

**[8] PAGE: Cimzia or certolizumab pegol**

This page asks about your use of Cimzia or certolizumab pegol to control your IBD symptoms.

81. Are you currently taking Cimzia® or certolizumab pegol?
    ( ) Yes
    ( ) No

    {IF ibd_certolizumab_curr_use = yes}
82. What is the longest period of time you took Cimzia® or certolizumab pegol without any interruption of more than 2 months?
    ( ) Less than 4 months
    ( ) 4 months - 1 year
    ( ) 1 - 5 years
    ( ) Over 5 years

    {IF ibd_certolizumab_curr_use = yes}
83. After using Cimzia® or certolizumab pegol for several months, how were your symptoms compared to your baseline?
    ( ) Symptoms got much worse
    ( ) Symptoms got a little worse
    ( ) Symptoms neither worsened nor improved
    ( ) Symptoms improved a little
    ( ) Symptoms improved a lot

    {IF ibd_certolizumab_curr_use = no}
84. How well did Cimzia® or certolizumab pegol work for you?
    ( ) Not at all
    ( ) A little
    ( ) Somewhat
    ( ) A fair amount
    ( ) A great deal

    {IF ibd_certolizumab_curr_use = yes}
85. How well does Cimzia® or certolizumab pegol work for you?
    ( ) Not at all
    ( ) A little
    ( ) Somewhat
    ( ) A fair amount
    ( ) A great deal

    {IF ibd_certolizumab_curr_use = no}
86. Why did you stop taking Cimzia® or certolizumab pegol?
    [ ] Side effects
    [ ] The medication did not control my IBD symptoms enough
    [ ] Cost or insurance coverage
    [ ] Other

    {IF ibd_certolizumab_curr_use = no}
87. Did you have any side effects while taking Cimzia® or certolizumab pegol?
    ( ) Yes
    ( ) No

    {IF diagnosed with Crohn’s or UC, AND ever took golimumab}

**[9] PAGE: Simponi or golimumab**

This page asks about your use of Simponi or golimumab to control your IBD symptoms.

88. Are you currently taking Simponi® or golimumab?
    ( ) Yes
    ( ) No

    {IF ibd_golimumab_curr_use = yes}
89. What is the longest period of time you took Simponi® or golimumab without any interruption of more than 2 months?
    ( ) Less than 4 months
    ( ) 4 months - 1 year
    ( ) 1 - 5 years
    ( ) Over 5 years

    {IF ibd_golimumab_curr_use = yes}
90. After using Simponi® or golimumab for several months, how were your symptoms compared to your baseline?
    ( ) Symptoms got much worse
    ( ) Symptoms got a little worse
    ( ) Symptoms neither worsened nor improved
    ( ) Symptoms improved a little
    ( ) Symptoms improved a lot

    {IF ibd_golimumab_curr_use = no}
91. How well did Simponi® or golimumab work for you?
    ( ) Not at all
    ( ) A little
    ( ) Somewhat
    ( ) A fair amount
    ( ) A great deal

    {IF ibd_golimumab_curr_use = yes}
92. How well does Simponi® or golimumab work for you?
    ( ) Not at all
    ( ) A little
    ( ) Somewhat
    ( ) A fair amount
    ( ) A great deal

    {IF ibd_golimumab_curr_use = no}
93. Why did you stop taking Simponi® or golimumab?
    [ ] Side effects
    [ ] The medication did not control my IBD symptoms enough
    [ ] Cost or insurance coverage
    [ ] Other

    {IF ibd_golimumab_curr_use = no}
94. Did you have any side effects while taking Simponi® or golimumab?
    ( ) Yes
    ( ) No

    {IF diagnosed with Crohn’s or UC, AND ever took natalizumab}
**[10] PAGE: Tysabri or natalizumab**

This page asks about your use of Tysabri or natalizumab to control your IBD symptoms.

95. Are you currently taking Tysabri® or natalizumab?
    ( ) Yes
    ( ) No

    {IF ibd_natalizumab_curr_use = yes}
96. What is the longest period of time you took Tysabri® or natalizumab without any interruption of more than 2 months?
    ( ) Less than 4 months
    ( ) 4 months - 1 year
    ( ) 1 - 5 years
    ( ) Over 5 years

    {IF ibd_natalizumab_curr_use = yes}
97. After using Tysabri® or natalizumab for several months, how were your symptoms compared to your baseline?
    ( ) Symptoms got much worse
    ( ) Symptoms got a little worse
    ( ) Symptoms neither worsened nor improved
    ( ) Symptoms improved a little
    ( ) Symptoms improved a lot

    {IF ibd_natalizumab_curr_use = no}
98. How well did Tysabri® or natalizumab work for you?
    ( ) Not at all
    ( ) A little
    ( ) Somewhat
    ( ) A fair amount
    ( ) A great deal

    {IF ibd_natalizumab_curr_use = yes}
99. How well does Tysabri® or natalizumab work for you?
    ( ) Not at all
    ( ) A little
    ( ) Somewhat
    ( ) A fair amount
    ( ) A great deal

    {IF ibd_natalizumab_curr_use = no}
100. Why did you stop taking Tysabri® or natalizumab?
    [ ] Side effects
    [ ] The medication did not control my IBD symptoms enough
    [ ] Cost or insurance coverage
    [ ] Other

    {IF ibd_natalizumab_curr_use = no}
101. Did you have any side effects while taking Tysabri® or natalizumab?
    ( ) Yes
    ( ) No

    {IF diagnosed with Crohn’s or UC, AND ever took ustekinumab}
**[11] PAGE: Stelara or ustekinumab**

This page asks about your use of Stelara or ustekinumab to control your IBD symptoms.

102. Are you currently taking Stelara® or ustekinumab?
    ( ) Yes
    ( ) No

    {IF ibd_ustekinumab_curr_use = yes}
103. What is the longest period of time you took Stelara® or ustekinumab without any interruption of more than 2 months?
    ( ) Less than 4 months
    ( ) 4 months - 1 year
    ( ) 1 - 5 years
    ( ) Over 5 years

    {IF ibd_ustekinumab_curr_use = yes}
104. After using Stelara® or ustekinumab for several months, how were your symptoms compared to your baseline?
    ( ) Symptoms got much worse
    ( ) Symptoms got a little worse
    ( ) Symptoms neither worsened nor improved
    ( ) Symptoms improved a little
    ( ) Symptoms improved a lot

    {IF ibd_ustekinumab_curr_use = no}
105. How well did Stelara® or ustekinumab work for you?
    ( ) Not at all
    ( ) A little
    ( ) Somewhat
    ( ) A fair amount
    ( ) A great deal

    {IF ibd_ustekinumab_curr_use = yes}
106. How well does Stelara® or ustekinumab work for you?
    ( ) Not at all
    ( ) A little
    ( ) Somewhat
    ( ) A fair amount
    ( ) A great deal

    {IF ibd_ustekinumab_curr_use = no}
107. Why did you stop taking Stelara® or ustekinumab?
    [ ] Side effects
    [ ] The medication did not control my IBD symptoms enough
    [ ] Cost or insurance coverage
    [ ] Other

    {IF ibd_ustekinumab_curr_use = no}
108. Did you have any side effects while taking Stelara® or ustekinumab?
    ( ) Yes
    ( ) No

    {IF diagnosed with Crohn’s or UC, AND ever took vedolizumab}
**[12] PAGE: Entyvio or vedolizumab**

This page asks about your use of Entyvio or vedolizumab to control your IBD symptoms.

109. Are you currently taking Entyvio® or vedolizumab?
    ( ) Yes
    ( ) No

    {IF ibd_vedolizumab_curr_use = yes}
110. What is the longest period of time you took Entyvio® or vedolizumab without any interruption of more than 2 months?
    ( ) Less than 4 months
    ( ) 4 months - 1 year
    ( ) 1 - 5 years
    ( ) Over 5 years

    {IF ibd_vedolizumab_curr_use = yes}
111. After using Entyvio® or vedolizumab for several months, how were your symptoms compared to your baseline?
    ( ) Symptoms got much worse
    ( ) Symptoms got a little worse
    ( ) Symptoms neither worsened nor improved
    ( ) Symptoms improved a little
    ( ) Symptoms improved a lot

    {IF ibd_vedolizumab_curr_use = no}
112. How well did Entyvio® or vedolizumab work for you?
    ( ) Not at all
    ( ) A little
    ( ) Somewhat
    ( ) A fair amount
    ( ) A great deal

    {IF ibd_vedolizumab_curr_use = yes}
113. How well does Entyvio® or vedolizumab work for you?
    ( ) Not at all
    ( ) A little
    ( ) Somewhat
    ( ) A fair amount
    ( ) A great deal

    {IF ibd_vedolizumab_curr_use = no}
114. Why did you stop taking Entyvio® or vedolizumab?
    [ ] Side effects
    [ ] The medication did not control my IBD symptoms enough
    [ ] Cost or insurance coverage
    [ ] Other

    {IF ibd_vedolizumab_curr_use = no}
115. Did you have any side effects while taking Entyvio® or vedolizumab?
    ( ) Yes
    ( ) No

    {IF diagnosed with Crohn’s or UC, AND ever took any salicylate}
**[13] PAGE: Salicylates**

This page asks about your use of salicylate medications to treat IBD. Salicylate medications include 5-aminosalicylic acid / 5-ASA, balsalazide / Colazal, Colazide, MMX, olsalazine / Dipentum, sulfasalazine / Azulfidine, Salazopyrin, Sulazine.

116. Are you currently taking a salicylate medication?
    ( ) Yes
    ( ) No

    {IF salicylates_curr_use = yes}
117. What is the longest period of time you took any salicylate medication without any interruption of more than 2 months?
    ( ) Less than 4 months
    ( ) 4 months - 1 year
    ( ) 1 - 5 years
    ( ) Over 5 years

    {IF salicylates_curr_use = yes}
118. After using your first salicylate medication for several months, how were your symptoms compared to your baseline?
    ( ) Symptoms got much worse
    ( ) Symptoms got a little worse
    ( ) Symptoms neither worsened nor improved
    ( ) Symptoms improved a little
    ( ) Symptoms improved a lot

    {IF salicylates_curr_use = no}
119. How well did the salicylate medications that you tried work for you overall?
    ( ) Not at all
    ( ) A little
    ( ) Somewhat
    ( ) A fair amount
    ( ) A great deal

    {IF salicylates_curr_use = yes}
120. How well do the salicylate medications that you tried work for you overall?
    ( ) Not at all
    ( ) A little
    ( ) Somewhat
    ( ) A fair amount
    ( ) A great deal

    {IF salicylates_curr_use = no}
121. Why did you stop taking your last salicylate medication? 
    [ ] Side effects
    [ ] The medication did not control my IBD symptoms enough
    [ ] Cost or insurance coverage
    [ ] Other

    {IF salicylates_curr_use = no}
122. Did you have any side effects while taking any salicylate medication?
    ( ) Yes
    ( ) No

    {IF diagnosed with Crohn’s or UC, AND ever took any corticosteroid}

**[14] PAGE: Corticosteroids**

This page asks about your use of corticosteroid medications to treat IBD. Corticosteroid medications include prednisone / Prednisolone, Deltasone, Winpred, budesonide / Entocort, methylprednisolone / Medrol, Meprolone, SoluMedrol, hydrocortisone / Colocort, Cortef, Cortenema, Cortifoam, Cortisol, SoluCortef, betamethasone / Betnesol, Betnovate, Celestone, dexamethasone, dexamethasone acetate, Dexacen LA-8, Dexasone, Dexpak.

123. Are you currently taking a corticosteroid medication?
    ( ) Yes
    ( ) No

    {IF corticosteroids_curr_use = yes}
124. What is the longest period of time you took any corticosteroid medication without any interruption of more than 2 months?
    ( ) Less than 4 months
    ( ) 4 months - 1 year
    ( ) 1 - 5 years
    ( ) Over 5 years

    {IF corticosteroids_curr_use = yes}
125. After using your first corticosteroid medication for several months, how were your symptoms compared to your baseline?
    ( ) Symptoms got much worse
    ( ) Symptoms got a little worse
    ( ) Symptoms neither worsened nor improved
    ( ) Symptoms improved a little
    ( ) Symptoms improved a lot

    {IF corticosteroids_curr_use = no}
126. How well did the corticosteroid medications that you tried work for you overall?
    ( ) Not at all
    ( ) A little
    ( ) Somewhat
    ( ) A fair amount
    ( ) A great deal

    {IF corticosteroids_curr_use = yes}
127. How well do the corticosteroid medications that you tried work for you overall?
    ( ) Not at all
    ( ) A little
    ( ) Somewhat
    ( ) A fair amount
    ( ) A great deal

    {IF corticosteroids_curr_use = no}
128. Why did you stop taking your last corticosteroid medication? 
    [ ] Side effects
    [ ] The medication did not control my IBD symptoms enough
    [ ] Cost or insurance coverage
    [ ] Other

    {IF corticosteroids_curr_use = no}
129. Did you have any side effects while taking any corticosteroid medication?
    ( ) Yes
    ( ) No

    {IF diagnosed with Crohn’s or UC, AND ever took any immunosuppressant}

**[15] PAGE: Immunosuppressants**

This page asks about your use of immunosuppressant medications to treat IBD. Immunosuppressant medications include azathioprine / AZA, Azasan, Imuran, 6-MP, mercaptopurine, Purinethol, methotrexate / MTX, Rheumatrex.

130. Are you currently taking an immunosuppressant medication?
    ( ) Yes
    ( ) No

    {IF immunosuppressants_curr_use = yes}
131. What is the longest period of time you took any immunosuppressant medication without any interruption of more than 2 months?
    ( ) Less than 4 months
    ( ) 4 months - 1 year
    ( ) 1 - 5 years
    ( ) Over 5 years

    {IF immunosuppressants_curr_use = yes}
132. After using your first immunosuppressant medication for several months, how were your symptoms compared to your baseline?
    ( ) Symptoms got much worse
    ( ) Symptoms got a little worse
    ( ) Symptoms neither worsened nor improved
    ( ) Symptoms improved a little
    ( ) Symptoms improved a lot

    {IF immunosuppressants_curr_use = no}
133. How well did the immunosuppressant medications that you tried work for you overall?
    ( ) Not at all
    ( ) A little
    ( ) Somewhat
    ( ) A fair amount
    ( ) A great deal

    {IF immunosuppressants_curr_use = yes}
134. How well do the immunosuppressant medications that you tried work for you overall?
    ( ) Not at all
    ( ) A little
    ( ) Somewhat
    ( ) A fair amount
    ( ) A great deal

    {IF immunosuppressants_curr_use = no}
135. Why did you stop taking your last immunosuppressant medication? 
    [ ] Side effects
    [ ] The medication did not control my IBD symptoms enough
    [ ] Cost or insurance coverage
    [ ] Other

    {IF immunosuppressants_curr_use = no}
136. Did you have any side effects while taking any immunosuppressant medication?
    ( ) Yes
    ( ) No

    {IF diagnosed with Crohn’s or UC, AND ever took any antibiotic to treat IBD}

**[16] PAGE: Antibiotics**

This page asks about your use of antibiotic medications to treat IBD. Antibiotic medications include metronizadole, ciproflaxin, rifaximin, clarithromycin / Biaxin.

137. Are you currently taking an antibiotic medication?
    ( ) Yes
    ( ) No

    {IF antibiotics_curr_use = yes}
138. What is the longest period of time you took any antibiotic medication without any interruption of more than 2 months?
    ( ) Less than 4 months
    ( ) 4 months - 1 year
    ( ) 1 - 5 years
    ( ) Over 5 years

    {IF antibiotics_curr_use = yes}
139. After using your first antibiotic medication for several months, how were your symptoms compared to your baseline?
    ( ) Symptoms got much worse
    ( ) Symptoms got a little worse
    ( ) Symptoms neither worsened nor improved
    ( ) Symptoms improved a little
    ( ) Symptoms improved a lot

    {IF antibiotics_curr_use = no}
140. How well did the antibiotic medications that you tried work for you overall?
    ( ) Not at all
    ( ) A little
    ( ) Somewhat
    ( ) A fair amount
    ( ) A great deal

    {IF antibiotics_curr_use = yes}
141. How well do the antibiotic medications that you tried work for you overall?
    ( ) Not at all
    ( ) A little
    ( ) Somewhat
    ( ) A fair amount
    ( ) A great deal

    {IF antibiotics_curr_use = no}
142. Why did you stop taking your last antibiotic medication? 
    [ ] Side effects
    [ ] The medication did not control my IBD symptoms enough
    [ ] Cost or insurance coverage
    [ ] Other

    {IF antibiotics_curr_use = no}
143. Did you have any side effects while taking any antibiotic medication?
    ( ) Yes
    ( ) No
